# Long-term functional donor site morbidity of the free radial forearm flap in head and neck cancer survivors

**DOI:** 10.1186/1916-0216-43-1

**Published:** 2014-01-13

**Authors:** Jason R Orlik, Peter Horwich, Clark Bartlett, Jonathan Trites, Robert Hart, S Mark Taylor

**Affiliations:** 1 Faculty of Medicine – Dalhousie University, Halifax, Nova Scotia, Canada; 2 Department of Surgery, Division of Otolaryngology, QEII Health Sciences Centre, Faculty of Medicine, Dalhousie University, Suite 3044 – Dickson Building, 5820 University Avenue, Halifax B3H 1 V7 NS, Canada

**Keywords:** Radial forearm flap, Donor-site, Morbidity, Long-term

## Abstract

**Background:**

To assess the functional donor site morbidity of the forearm free flap in patients surviving at least 2 years after ablative head and neck cancer surgery in a tertiary care centre.

**Methods:**

This study involved nine long-term survivors (2 year post-operative) who had forearm free flaps to reconstruct head and neck defects. All flaps were raised from the non-dominant arm. The non-donor side acted as a control for all patients. Objective measurements were as follows: grip, tip pinch and key pinch strength measured with dynamometers; flexion, extension, radial and ulnar deviation and pronation and supination range of motion at the wrist measured with goniometry; A timed manual dexterity task was performed with a grooved pegboard test, and sensation of the radial nerve was tested with Semmes Weinstein monofilaments. Subjective measurements included a validated patient questionnaire of hand function and opinions of scar appearance as well as a validated scar assessment from two different observers.

**Results:**

Pronation at the wrist, manual dexterity and sensation were found to be significantly reduced in the donor side compared to the non-donor side. Inter-rater agreement between the two observers was found to be poor, except for an acceptable correlation between overall scar opinions. No correlations were found between any subjective or objective items or between the patient’s and the observers’ subjective evaluations.

**Conclusions:**

Donor site morbidity can be demonstrated with objective testing however this is accepted and well tolerated by head and neck cancer patients.

## Introduction

The forearm flap is a reliable and versatile method to reconstruct various structures in the head and neck [1-3]. It remains the workhorse free flap for head and neck defects [2,4]. There are several reasons for its current popularity: 1) The presence of a long vascular pedicle of adequate caliber; 2) Pliability and thinness allow for complex reconstruction; and 3) and the anatomic location of the flap allows for simultaneous harvest with the ablative team [5,6].

Much attention in the literature has addressed the success of the forearm flap for various head and neck defects [1-4]. There continues to be controversy over the morbidity of the donor site. Potential complications include: decreased range of motion in the wrist, reduced hand strength, diminished sensation, painful neuroma, skin graft loss over donor site resulting in tendon exposure, numbness, itching, cold intolerance, poor aesthetic appearance, limited finger range of motion, and delayed healing [2,5-17].

Some previous studies have found statistically significant functional morbidity [15]. In order to prevent these, various skin-grafting techniques to the donor site, post operative wound site care, and modified flaps or different donor sites have been advocated [6,8,12]. Few studies have reported on the patients’ subjective views of the donor site satisfaction [6,14,18]. Also, there are very few studies reporting follow up regarding donor site morbidity in long term survivors [6,7]. Hand sensation, grip strength and range of motion at the wrist are important functions in active, daily life, and compromise can have profound effects on the patient's quality of life.

## Materials and methods

**Figure 1:**
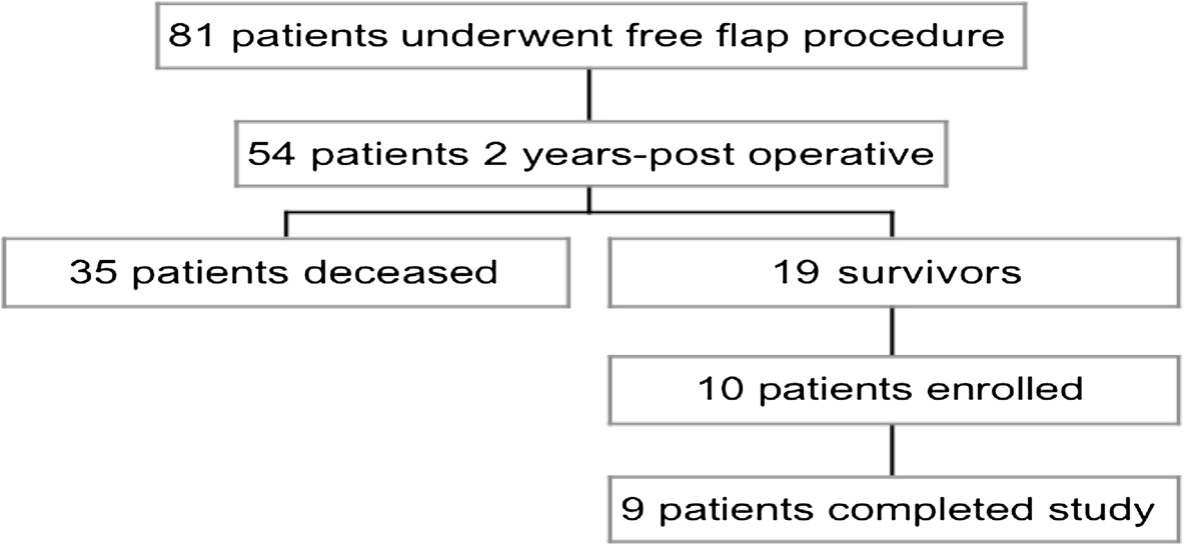
Patient breakdown.

Capital Health Research Ethics Board granted approval of this study. Nineteen individuals were identified as having a radial forearm free flap (RFFF) for head and neck reconstruction at least two years post cancer resection. Ten patients who met the inclusion criteria signed consent forms and were enrolled in the study, of which nine completed all aspects of the study (Figure 1). Allen’s Tests were performed to ensure adequate blood flow in the ulnar artery prior to surgery. The flaps were raised from the patient’s non-dominant arms. All patients had split-thickness skin graft of 16/1000-inch thickness, harvested from the thigh to reconstruct the forearm donor site. A standard bolster dressing was applied and a volar slab was left in place for one week. Exclusion criteria were problems of movement, strength, or sensation of the hands or forearms prior to operation.

### Evaluations

Validated patient and observer scar assessment questionnaires developed by van de Kar et al. [19,20] were utilized, modified to include such items, among others, as activities of daily living; cold intolerance; wearing short-sleeved shirts and watches or bracelets on the donor side (Additional file 1). These are subjective rating scales of the patients’ perceptions of their forearm scar as well to observers. Two observers also used a questionnaire to evaluate the donor site (Additional file 2). Individual observers and participants were blinded to all other assessments of donor sites. For observers, blinding was ensured by completing their assessments individually - examining the donor site in one room and completing the questionnaire in a separate room. For participants, blinding was ensured by having them complete their donor site assessment alone, before the first observer arrived. All assessment forms were collected immediately after assessment – editing assessments after the initial scoring was not permitted.

The observers were a medical student and an otolaryngology senior resident.

A Jamar grip dynamometer (Sammons Preston, Bollingbrook, IL) was used to measure grip strength. Tip pinch strength and key grip strength were measured with a pinch gauge (Sammons Preston, Bollingbrook, IL). A stainless steel wrist goniometer was used to measure wrist flexion, extension, radial and ulnar deviation, and pronation and supination. Measurement protocols were taken from Solgaard et al. [21].

A timed grooved pegboard test evaluated dexterity (Lafayette Instrument, Lafayette, IN). Sensation was tested via Semmes Weinstein monofilaments applied to the radial nerve distribution over the scaphoid on the distal forearm.

We analyzed the results of this study using a Student’s t test, Pearson’s Correlation, and Cohen’s Kappa was used to return a measurement of inter-rater agreement between observers. Statistical analysis was performed using Microsoft Excel and statistical significance was defined as < 0.05.

## Results

**Figure 2:**
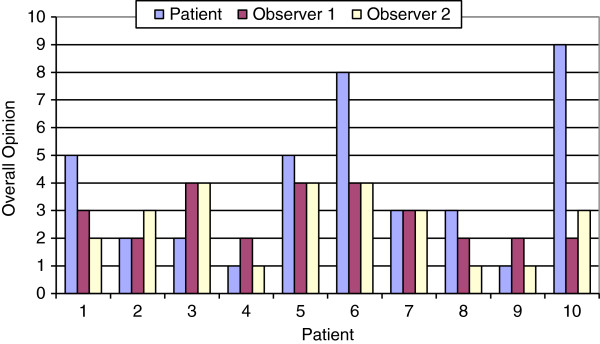
**Subjective scar rating, overall opinion.** Observer 1 = medical student. Observer 2 = otolaryngology resident. Overall opinion was represented on a likert scale 1 = no difference from un-operated arm, 10 = yes, very different.

**Figure 3:**
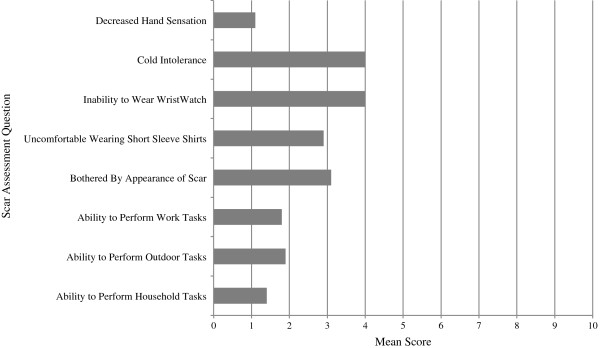
**Mean subjective scores from assessment scale questionnaire.** Mean subjective score was represented on a likert scale 1 = no difference from un-operated arm, 10 = yes, very different.

Patient demographic information for those enrolled in the study are illustrated in Table 1. There were no significant correlations between the patients overall opinion and the overall opinions of the observers (Figure 2). No correlation was identified between objective measurements and subjective ratings. No correlation was found between subjective or objective measurements and age, tumour stage, or length of post-operative time to testing. Mean scores from individual questions on the scar assessment scale questionnaire are presented in Figure 3.

**Table 1:** Patient demographics and post-operative time

**Characteristic**	**Value**
Patient Age Range (mean)	37 – 72 (59)
Male/Female Ratio	5:5
Months Post-Operative (mean)	26 – 109 (50)

Two patients had post-operative complications of the donor arm – one patient had a donor forearm fracture on post-operative day 2, the other patient had a stitch abscess. Only one patient reported difficulty in using the donor arm to perform household, outdoor, or work tasks. In patient reported aesthetics, 3 patients reported being bothered by the appearance of their scar and being uncomfortable wearing short sleeves in public. Three patients also reported the inability to wear a wristwatch on the donor arm, while four patients reported cold intolerance (Figure 3).

**Figure 4:**
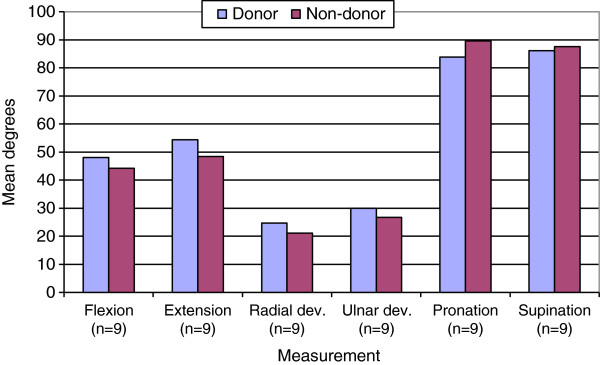
**Mean wrist range of motion.** Mean donor side pronation is significantly less (p = 0.031).

Mean wrist range of motion on the donor side exceeded that of the non-donor side for flexion (p = 0.21), extension (p = 0.31), radial (p = 0.47) and ulnar deviation (p = 0.25), though the difference was not significant (Figure 4). Mean percentages of the non-donor arm were greater than 110 percent. Using a Students paired t-test found mean pronation of the wrist on the donor side to be significantly less than the non-donor side (p = 0.031) (Table 2).

**Table 2:** Range of motion at the wrist (n = 9)

	**Donor**			**Non-donor**				**T-test**	
	**Mean (deg)**	**SD (deg)**	**95% C.I.**	**Mean (deg)**	**SD (deg)**	**95% C.I.**	**Mean diff (deg)**	**P-value (sig)**	**Mean % of non-donor arm**
Flexion (n = 9)	48.11	12.02	7.85	44.22	11.41	7.45	3.89	0.21	110.51
Extension (n = 9)	54.44	14.28	9.33	48.44	8.05	5.26	6.00	0.31	115.38
Radial Dev. (n = 9)	24.78	10.30	6.73	21.11	10.50	6.86	3.67	0.47	143.38
Ulnar Dev. (n = 9)	30.00	6.16	4.02	26.78	4.58	2.99	3.22	0.25	114.79
Pronation (n = 9)	83.89	8.08	5.28	89.56	8.14	5.32	-5.67	0.03	93.91
Supination (n = 9)	86.11	7.88	5.15	87.56	8.63	5.64	-1.45	0.71	99.11

**Figure 5:**
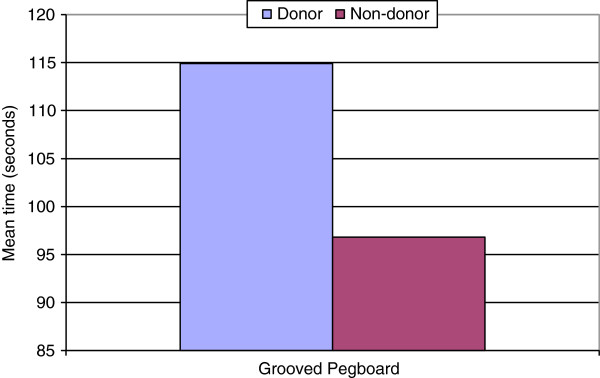
**Manual dexterity.** Mean grooved pegboard completion time was significantly slower on the donor side (p = 0.008).

**Figure 6:**
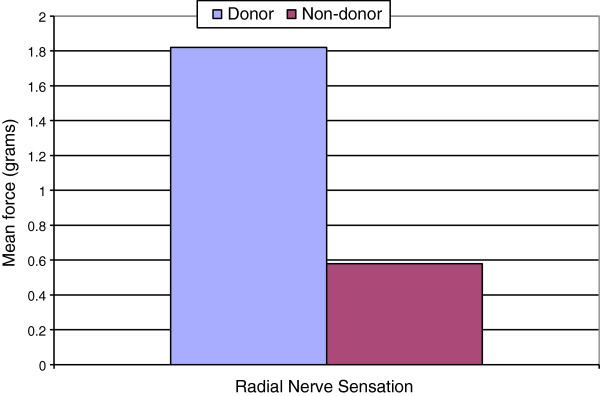
**Radial nerve sensation.** Mean radial nerve sensation significantly reduced on the donor side (p = 0.017).

All aspects of grip strength on the donor side were reduced compared to the non-donor side, however, this was not found to be statistically significant (p = 0.17-0.63). Mean percentages of the un-operated arm were all over 94%. An expected 10-15% reduction in strength on the non-dominant side was not observed (Table 3). Manual dexterity was significantly slower on the operated side than on the non-operated side (p = 0.008) (Figure 5). Mean percentage of the un-operated arm was 117.91%. Mean sensation of the radial nerve, as tested at the anatomical snuffbox over the scaphoid, was significantly decreased compared to the non-donor side (p = 0.017) (Figure 6). Mean percentage of the un-operated arm was 313.79 percent (Table 3). However, this did not coincide with sensation differences as reported by the patient questionnaires (Figure 3).

**Table 3:** Hand strength

	**Donor**			**Non-donor**				**T-test**	
	**Mean (Kg)**	**SD (Kg)**	**95% C.I.**	**Mean (Kg)**	**SD (Kg)**	**95% C.I.**	**Mean diff (Kg)**	**P-value (sig)**	**Mean % of non-donor arm**
Grip Strength (n = 10)	34.70	14.83	9.19	35.40	14.62	9.06	-0.70	0.63	98.82
Tip Pinch (n = 9)	3.36	2.06	1.35	4.23	2.82	1.84	-0.87	0.17	94.99
Key Pinch (n = 9)	4.48	1.86	1.22	4.72	2.10	1.37	-0.24	0.55	98.42
	Mean (Secs)	SD (Secs)	95% C.I.	Mean (Secs)	SD (Secs)	95% C.I.	Mean Diff (Secs)	P-Value (sig)	Mean % of Non-Donor Arm
Pegboard (n = 10)	114.90	45.21	28.02	96.80	33.22	20.59	18.10	0.01	117.91
	Mean (g)	SD (g)		Mean (g)	SD (g)	95% C.I.	Mean Diff (g)	P-Value (sig)	Mean % of Non-Donor Arm
Sensation (n = 9)	1.82	1.31	0.86	0.58	0.84	0.55	1.24	0.02	313.79

Cohen’s Kappa was used to return a measurement of inter-rater agreement between observer 1 (a medical student) and observer 2 (a senior otolaryngology resident). Inter-rater agreement was found to be poor throughout all categories of evaluation with Cohen’s Kappa values of 0.50, 0.26, 0, 0.46, and -0.07 for the first five questions respectively. However, use of Pearson’s Correlation found acceptable Pearson’s Correlation value of 0.78 for overall opinion of patient scars between the two observers. A weaker correlation was noted between the total scar assessment scores of observer 1 and total patient questionnaire scores with a Pearson’s Correlation value of 0.69.

## Discussion

Although donor site morbidity was demonstrated objectively, this did not coincide with subjective patient reporting. This study attempted to address the lack of literature regarding radial forearm flap donor site morbidity in long-term head and neck cancer survivors. An unfortunate limitation of this study was a small sample size. Absence of non-donor arm evaluated as an internal control is cited as a limitation in previous studies [15]. Without pre-operative data, the non-donor arm is very useful. However, studies have shown a 10% difference in strength between dominant and non-dominant hands [22,23]. A 10 percent reduction in grip strength was therefore expected in our patients, as all donor arms were also the non-dominant arms. It is interesting to note that donor arms were reduced only 2–4 percent from the non-donor arm. This coincides with a study by Ho et al. [6] who in forearm flap patients with a median follow-up of 51 months. They observed a 91–98 median percentage in strength of the un-operated arm [6]. A longitudinal study of people over 65 found that grip strength declines 2% per year for men and women. This was attributed to lack of use rather than ill health [22]. Limb dominance was not considered for wrist goniometry. A previous study found that the opposite wrist serves as a reference for evaluating motion restrictions, as there was no significant difference in wrist angles between left and right. It was also determined that no significant difference existed between experienced and non-experienced observers [21]. Therefore, the fact that all but one of the wrist ranges of motion measurements is insignificantly greater is unsurprising. A previous study found range of motion of the donor arm as a median percentage of the un-operated arm to range from 92-117%. Pronation was, however, found to be significantly less on the donor arm. A previous forearm flap study found a decreased wrist extension of less than 5 degrees compared to the non operated side in 6 patients [17]. An early post-operative study demonstrated significant morbidities of supination, pronation, flexion, extension and sensation within the anatomical snuffbox when compared to pre-operative performance [15].

Sensation over the anatomical snuffbox was significantly reduced compared to the un-operated arm, although there was no correlation with sensation as reported in the patients’ questionnaires. Dexterity of the donor arm as determined by the grooved pegboard test was also significantly less than the non-donor side. Due to the donor arm also being the non-dominant arm, it is expected that a portion of the time difference in dexterity will be due to the use of the dominant hand versus the non-dominant hand. Pre-operative donor hand dexterity testing to compare to post-operative donor hand dexterity was not completed and is a limitation of this study. Normative values for this age range have found a mean difference of 106% [23]. However, this study displayed a difference of over 300%. This could be due to outliers in the small sample size included in this study. However, despite this objective measurement of difference in dexterity, only one patient reported a difference in their ability to adequately perform tasks at work, in the household or outdoors.

Patient demographic information for those enrolled in the study are illustrated in Table 1. A modified version of the patient and observer scar assessment scale was utilized in this study. The patient and observer scar assessment scale was chosen over the more popular Vancouver scar assessment scale as it accounts for the opinion of the patient as well as having better reliability [19,20]. Two patients encountered post-operative complications of the donor site – one had a forearm fracture and the other a stitch abscess. These complications may have impacted scar formation and subsequently altered subjective scar satisfaction. There was no correlation found with any component of the subjective evaluations with that of the objective measurements. Poor inter-rater agreement between the two observers suggests the highly subjective nature of scar assessment. However, one observer was a medical student while the other was a senior otolaryngology resident. Therefore, a portion of the poor inter-rater agreement of scar assessment may be due to the expertise disparity between observers. In a study comparing full and partial thickness skin grafts at the donor site, no significant difference was found between the two groups in terms of patient assessment of aesthetic appearance or pain in the donor site [24]. One study found no significant evidence of objective morbidity, while subjective complaints were numerous, with conclusion that more elaborate pre-surgical counseling would reduce this [14]. There are several established variations on the forearm flap. Pre-laminated fasciocutaneous flaps can reduce donor site morbidity of wrist extension, hand strength and sensation, and improved cosmesis [17]. Split thickness skin grafts have a non-significantly better outcome score as evaluated by surgeons, while the full thickness skin graft had slightly decreased wrist flexion and ulnar deviation [6].

## Conclusion

The forearm flap remains a reliable and versatile option in head and neck reconstruction. However, objective and subjective concerns regarding the donor site post-operatively must be considered prior to surgery. This study has shown that while objective testing can demonstrate donor site morbidity, subjective testing reveals that overall patients are functionally satisfied and tolerate the donor site post-operatively.

## Competing interests

At the time of the research, the authors had no conflicts of interest to disclose.

## Authors' contributions

JO was responsible for study design, data collection, statistical analysis and manuscript preparation. PH was responsible for manuscript preparation. CB, JT, and RH were involved in study design, data collection, and interpretation of results. SMT is the corresponding author and was involved in study design, data collection, interpretation of results and manuscript preparation. All authors read and approved the final manuscript.

## Supplementary Material

Additional file 1 **Patient Scar Assessment Scale modified from scar assessment scale by van de Kar et al.**[19,20]. Click here for file

Additional file 2 **Observer Scar Assessment Scale from scar assessment scale by van de Kar et al.**[19,20]. Click here for file
