# Supplementary material for: Long-term functional donor site morbidity of the free radial forearm flap in head and neck cancer survivors
Source: J Otolaryngol Head Neck Surg. 2014 Jan 13;43(1):1. doi: 10.1186/1916-0216-43-1 (PMC3895707; doi:10.1186/1916-0216-43-1)
Supplement: Additional file 1 — Patient Scar Assessment Scale modified from scar assessment scale by van de Kar et al.[19,20]. [file 1916-0216-43-1-S1.doc]

Additional file 1. ­Patient Scar Assessment Scale modified from scar assessment scale by van de Kar et al^19 20^.

| Please answer the following questions about your forearm using the scale below    example  No, no complaints 1 2 3 4 5 6 7 8 9 10 Worst imaginable   1. Has the scar been painful the past few weeks?   1 2 3 4 5 6 7 8 9 10   1. Has the scar been itching the past few weeks?   1 2 3 4 5 6 7 8 9 10  Please answer the following questions about your forearm using the scale below  example  No, as normal skin 1 2 3 4 5 6 7 8 9 10 Yes, very different   1. Is the scar colour different from the colour of your normal skin present?   1 2 3 4 5 6 7 8 9 10   1. Is the stiffness of the scar different from your normal skin at present?   1 2 3 4 5 6 7 8 9 10   1. Is the thickness of the scar different from your normal skin at present?   1 2 3 4 5 6 7 8 9 10   1. Is the scar more irregular than your normal skin at present?   1 2 3 4 5 6 7 8 9 10  7) What is your overall opinion of the scar compared to normal skin?   1. 2 3 4 5 6 7 8 9 10 2. I have heightened sensitivity in my hand   1 2 3 4 5 6 7 8 9 10   1. I have heightened sensitivity in my forearm   1 2 3 4 5 6 7 8 9 10  10) I have decreased hand sensitivity  1 2 3 4 5 6 7 8 9 10  11) I have decreased sensitivity in forearm  1 2 3 4 5 6 7 8 9 10  12) I have noticed a change in my ability to perform household tasks  1 2 3 4 5 6 7 8 9 10  13) I have noticed a change in my ability to perform outdoor tasks  1 2 3 4 5 6 7 8 9 10  14) I have noticed a change in my ability to perform work tasks  1 2 3 4 5 6 7 8 9 10  15) I am bothered by the appearance of my forearm  1 2 3 4 5 6 7 8 9 10  16) I am uncomfortable with wearing short sleeved shirts  1 2 3 4 5 6 7 8 9 10  17) I cannot wear a wristwatch or bracelet on the side of the operation  1 2 3 4 5 6 7 8 9 10    18) My hand feels numb  1 2 3 4 5 6 7 8 9 10  19) The scar feels sore  1 2 3 4 5 6 7 8 9 10  20) I experience problems in the cold  1 2 3 4 5 6 7 8 9 10  Total Score (Patient Scar Scale) |
| --- |
