# Supplementary material for: Long-term functional donor site morbidity of the free radial forearm flap in head and neck cancer survivors
Source: J Otolaryngol Head Neck Surg. 2014 Jan 13;43(1):1. doi: 10.1186/1916-0216-43-1 (PMC3895707; doi:10.1186/1916-0216-43-1)
Supplement: Additional file 2 — Observer Scar Assessment Scale from scar assessment scale by van de Kar et al.[19,20]. [file 1916-0216-43-1-S2.doc]

Additional file 2. ­Observer Scar Assessment Scale from scar assessment scale by van de Kar et al^19 20^.

| Observer Scar Assessment Scale  Normal skin 1 2 3 4 5 6 7 8 9 10 Worst scar imaginable  1) Vascularity  1 2 3 4 5 6 7 8 9 10  Pale  Pink  Red  Purple  Mix    2) Pigmentation  1 2 3 4 5 6 7 8 9 10  Hypo  Hyper  Mix  3) Thickness  1 2 3 4 5 6 7 8 9 10  Thicker  Thinner  4) Relief  1 2 3 4 5 6 7 8 9 10    More relief  Less relief  mix  5) Pliability  1 2 3 4 5 6 7 8 9 10    Supple  Stiff  Mix  6) Normal skin 1 2 3 4 5 6 7 8 9 10 Worst scar imaginable  7) Overall opinion 1 2 3 4 5 6 7 8 9 10  Total Score (Observer Scar Scale) |
| --- |
